# Comparison of Whole-Genome Sequences of *Legionella pneumophila* in Tap Water and in Clinical Strains, Flint, Michigan, USA, 2016

**DOI:** 10.3201/eid2511.181032

**Published:** 2019-11

**Authors:** Emily Garner, Connor L. Brown, David Otto Schwake, William J. Rhoads, Gustavo Arango-Argoty, Liqing Zhang, Guillaume Jospin, David A. Coil, Jonathan A. Eisen, Marc A. Edwards, Amy Pruden

**Affiliations:** West Virginia University, Morgantown, West Virginia, USA (E. Garner);; Virginia Polytechnic Institute and State University, Blacksburg, Virginia, USA (E. Garner, C.L. Brown, W.J. Rhoads, G. Arango-Argoty, L. Zhang, M.A. Edwards, A. Pruden);; Methodist University, Fayetteville, North Carolina, USA (D.O. Schwake);; University of California at Davis, Davis, California, USA (G. Jospin, D.A. Coil, J.A. Eisen)

**Keywords:** Legionella, Legionnaires’ disease, drinking water, tap water, whole-genome sequencing, Flint, Michigan, United States, Legionella pneumophila, bacteria

## Abstract

During the water crisis in Flint, Michigan, USA (2014–2015), 2 outbreaks of Legionnaires’ disease occurred in Genesee County, Michigan. We compared whole-genome sequences of 10 clinical *Legionella pneumophila* isolates submitted to a laboratory in Genesee County during the second outbreak with 103 water isolates collected the following year. We documented a genetically diverse range of *L. pneumophila* strains across clinical and water isolates. Isolates belonging to 1 clade (3 clinical isolates, 3 water isolates from a Flint hospital, 1 water isolate from a Flint residence, and the reference Paris strain) had a high degree of similarity (2–1,062 single-nucleotide polymorphisms), all *L. pneumophila* sequence type 1, serogroup 1. Serogroup 6 isolates belonging to sequence type 2518 were widespread in Flint hospital water samples but bore no resemblance to available clinical isolates. *L. pneumophila* strains in Flint tap water after the outbreaks were diverse and similar to some disease-causing strains.

Legionnaires’ disease is a severe form of pneumonia caused by inhalation of virulent species of aerosolized *Legionella* bacteria. In January 2016, the Michigan Department of Health and Human Services (MDHHS) and the Genesee County Health Department publicly announced 2 Legionnaires’ disease outbreaks in Genesee County, Michigan, USA (*1*,*2*). The first outbreak occurred from June 2014 through March 2015 and the second from May 2015 through October 2015; a total of 90 cases and 12 deaths were documented (*1*–*3*). From April 2014 through October 2015, the city of Flint, in Genesee County, switched its drinking water source from Detroit Water and Sewer Department (DWSD), which used corrosion control, to the corrosive Flint River, without implementing federally mandated corrosion control; this new water source led to elevated lead in tap water over a prolonged period, now called the Flint water crisis (*4*). This disruption in water quality likely also stimulated the growth of *L. pneumophila*, the species most frequently identified as the causative agent of Legionnaires’ disease (*5*,*6*), in Flint’s distribution and plumbing systems (*7*).

Our prior work associated the Legionnaires’ disease outbreaks with factors known to be conducive to *Legionella* growth: elevated iron (a consequence of corroded iron water mains), reduced free chlorine disinfectant residuals, and elevated water temperatures (*7*,*8*). Later, Zahran and colleagues reported that the odds of Flint residents being referred for Legionnaires’ disease treatment while the Flint River was the source of tap water increased 6.3-fold and confirmed our report of associations with low chlorine residuals (*9*), but the odds analysis, which was based on the use of referral date rather than symptom onset date, excluded many healthcare-associated cases (*10*). Furthermore, during the second outbreak, *Legionella* spp. and *L. pneumophila* genes were found to be higher in the tap water of large buildings in Flint than in other water systems in US areas not experiencing outbreaks (*8*). Conversely, levels of the *mip* gene, which is specific to *L. pneumophila*, were largely below detection in Flint single-family residences, at least during the later stages of the water crisis when they were measured (2015–2016) (*8*). Large buildings with extensive plumbing networks, such as hospitals, are generally more susceptible to *Legionella* growth than are simpler plumbing systems characteristic of single-family homes (*11*); however, residences are also of interest for *Legionella* growth, given concerns about the high rate of sporadic Legionnaires’ disease (*12*) and potential for exposure in the home.

Our study objective was to use next-generation DNA sequencing to compare *L. pneumophila* isolated from Flint tap water after the second Legionnaires’ disease outbreak with tap water isolates from neighboring drinking water systems outside of Flint that were never served by Flint River water and clinical strains received during the second outbreak at a regional reference laboratory in Genesee County. Within Flint, *Legionella* isolates were obtained from the tap water of a hospital, a large public building, and single-family residences several months after the water source was switched back to DWSD. In addition to serogroup testing, we used whole-genome sequencing to compare isolates in terms of sequence type (ST), average nucleotide identity, and single-nucleotide polymorphisms (SNPs).

## Materials and Methods

### Water Sample Collection and *Legionella* Isolation

After Flint resumed purchasing water with corrosion control from the original supplier, DWSD, water sampling campaigns were conducted 5 months (March 7–9, 2016), 8 months (June 21–27, 2016), and 10 months (August 15–16, 2016) later. Samples were collected from residences, small businesses, a large public building, and a hospital in Flint; as controls, samples were collected from buildings located outside of Flint that used DWSD or well water (Table 1). The March 2016 campaign targeted sampling of residences, small businesses, a large public building, and a hospital; samples were collected from hot (flushed for 30 seconds) and cold (stagnant) taps at each location. Samples were collected from the kitchen sink in homes and from restrooms in public buildings. The June 2016 campaign extensively sampled homes as part of a water heater cleaning campaign; the following samples were collected before and after a cleaning protocol: hot and cold stagnant kitchen tap samples, a stagnant shower sample of blended hot and cold water, a hot flushed kitchen tap sample, the water heater drain valve, and a flushed cold water sample from the outside hose bib or nearest tap to the service entry point. The August 2016 campaign targeted sampling from hot (flushed 30 seconds) and cold (stagnant) water taps from homes and small businesses. *Legionella* was cultured according to standard methods (*13*), and colonies were streaked to isolation.

**Table 1:** Total number of buildings sampled, number of samples collected, and number of isolates analyzed for *Legionella*, Flint, Michigan, USA*

Water sample source	March 2016				June 2016				August 2016		
	No. buildings or samples	No. (%) positive	No. isolates analyzed		No. buildings or samples	No. (%) positive	No. isolates analyzed		No. buildings or samples	No. (%) positive	No. isolates analyzed
Flint residences	**5**	**0**			**32**†	**2 (6)**			**10**‡	**2 (20)**	
Hot (flushed)	5	0	0		62	2 (3)	3		14	1 (7)	1
Hot (stagnant)	NS				62	2 (3)	4		NS		
Cold (flushed)	NS				61	1 (2)	2		NS		
Cold (stagnant)	5	0	0		61	1 (2)	4		11	4 (36)	1
Water heater drain valve	NS				62	1 (2)	5		NS		
Shower (hot and cold)	NS				62	1 (2)	2		3	1 (33)	1
Hospitals	**1**	**1 (100)**			**NS**				**NS**		
Hot (flushed)	19	16 (84)	56		NS				NS		
Cold (stagnant)	19	6 (32)	14		NS				NS		
Buildings receiving DWSD water	**4**	**0**			**NS**				**8**	**0**	
Hot (flushed)	4	0	0		NS				8	0	0
Cold (stagnant)	4	0	0		NS				8	0	0
Flint large buildings	**2**	**1 (50)**			**NS**				**NS**		
Hot (flushed)	5	0	0		NS				NS		
Cold (stagnant)	5	1 (20)	1		NS				NS		
Buildings receiving well water	**1**	**1 (100)**			**NS**				**NS**		
Hot (flushed)	4	4 (100)	5§		NS				NS		
Cold (stagnant)	3	2 (67)	4¶		NS				NS		
Flint small businesses	**6**	**0**			**NS**				**8**	**0**	
Hot (flushed)	6	0	0		NS				8	0	0
Cold (stagnant)	6	0	0		NS				8	0	0

*Positive samples indicate presumptive *L. pneumophila* identified by performing culture according to the method described in (*12*). Unless otherwise noted, identification as *L. pneumophila* was confirmed by using whole-genome sequencing. Boldface indicates total buildings sampled. Blank cells indicate that data were not reported when applicable samples were not collected. NS, no samples of this type were collected.  †1 of the 32 homes was also sampled in March 2016. ‡5 of the 10 homes were also sampled in March 2016; 1 of the 10 was sampled in June 2016 (but not in March 2016; samples from this house were positive on both dates). §4 of 5 isolates were a non–*L. pneumophila* species, according to whole-genome sequencing. ¶4 of 4 isolates were a non–*L. pneumophila* species, according to whole-genome sequencing.

### Clinical Isolates

MDHHS provided 11 clinical isolates from de-identified Legionnaires’ patients who received a diagnosis in 2015; however, 1 isolate could not be cultured and was deemed nonviable. When we initiated this study, we assumed that all 11 isolates originated from patients with some history of exposure in Flint or Genesee County during the Flint water crisis. However, we later learned that the commonality among clinical isolates was that they had been submitted to a Genesee County laboratory for analysis during the second outbreak and that 3 of the 11 isolates originated from patients who resided and received treatment outside of Genesee County (J. McFadden, MDHHS, pers. comm., 2017 Feb 1). Because the clinical isolates in this study were de-identified, comparison with the water isolates is described in terms of “*L. pneumophila* known to be capable of causing LD.” We also included publicly available DNA sequence information from clinical reference strains in the analysis (Appendix 1 Table 2).

### Whole-Genome Analysis of *L. pneumophila* Isolates

Whole-genome sequencing was conducted by MicrobesNG (https://microbesng.uk) on an Illumina MiSeq (https://www.illumina.com) with 2 × 250-bp paired-end reads and Nextera library preparation (Illumina). Sequencing was performed for a representative subset of each building type and water source, including 103 water isolates and the 10 available clinical *L. pneumophila* isolates (Appendix 1 Table 1). To verify DNA integrity, DNA extracts were quantified via a Qubit 2.0 Fluorometer (https://www.thermofisher.com) and analyzed via gel electrophoresis. Positive (*L. pneumophila* strain 130b) and negative (*Stenotrophomonas maltophilia*) control strains were also sequenced, and 3 clinical strains were sequenced in duplicate on 2 MiSeq runs to evaluate run-to-run variation (Appendix 1 Figures 1–3). On average, 806,825 reads were obtained per isolate (range 280,380–2,031,828 reads). Reads were trimmed by using Trimmomatic (*14*), and de novo assemblies were generated by using SPAdes (*15*).

Genome sequences are available in GenBank under BioProject PRJNA453403. *Legionella* species assignments were determined via blastn (https://blast.ncbi.nlm.nih.gov) for isolate 16S rRNA gene sequences. Average nucleotide identity was calculated as previously described (*16*), and SNPs were identified by using kSNP3.0 (*17*). We also included 9 *L. pneumophila* reference strains (Appendix 1 Table 2). We performed sequence-based typing targeting the *flaA*, *pilE*, *asd*, *mip*, *mompS*, *proA*, and *neuA* alleles (*18*) by using the mompS tool (*19*).

### Serogroup Analysis

We identified *L. pneumophila* isolates belonging to serogroup 1 via detection of the *wzm* gene (*20*) in whole genome sequences. We verified DNA sequence-based classifications and determined unknown serogroups by using direct fluorescent antibody staining with fluorescein isothiocyanate–conjugated antibodies (m-TECH, http://www.4m-tech.com).

## Results

### *Legionella* Isolate Characterization

Of the 515 total water samples collected and from which *L. pneumophila* isolation was attempted (Table 1), 43 samples (8%) were positive for *Legionella*. Of these, 22 (58%) of 38 hospital samples from March 2016, eight (2%) of 370 residence samples from June 2016 (positives originating from 2 separate residences), and 6 (21%) of 28 residence samples from August 2016 (positives originating from different taps in a single residence) were positive for culturable *L. pneumophila*. No isolates were obtained from businesses receiving DWSD water, but 6 (86%) of 7 taps at the school serviced by well water were identified as positive (although 5 of these were later determined to be *Legionella* species other than *L.* *pneumophila*).

16S rRNA genes mined from whole-genome sequences indicated that all clinical and water isolates, except for 8 of the 9 well water isolates, were *L. pneumophila*. The positive control strain was correctly identified as *L. pneumophila*; SNP analysis further classified it according to its known provenance (130b), and the negative control strain was also confirmed to be *S. maltophilia* (i.e., not *Legionella*). Serogrouping via presence of the *wzm* gene for serogroup 1 and direct fluorescent antibody staining for other serogroups indicated that all *L. pneumophila* isolates belonged to serogroups 1 and 6 (Table 2).

**Table 2:** Summary of *Legionalla pneumophila* isolates from Flint, Michigan, USA, 2016

ST	Serogroup	Isolate origin*
1	1	3 hospital water (HH17, HH25, HH56), 1 residence water (RH08), 3 clinical (C2, C3, C7)
44	1	1 clinical (C6)
159	1	1 clinical (C1)
192	1	19 residence water (RC01, RC02, RC03, RC04, RC06, RC07, RD01, RD02, RD03, RD04, RD05, RH02, RH03, RH04, RH05, RH07 RH07, RS01, RS02)
211	1	1 clinical (C8)
213	1	2 clinical (C4, C5)
222	1	1 clinical (C9)
2513†	1	1 clinical (C10)
2518†	6	66 hospital water (HC01, HC02, HC03, HC04, HC05, HC06, HC07, HC08, HC09, HC10, HC11, HC12, HC13, HC14, HH01, HH02, HH03, HH04, HH05, HH06, HH07, HH08, HH09, HH10, HH11, HH12, HH13, HH14, HH15, HH16, HH18, HH19, HH20, HH21, HH22, HH23, HH24, HH26, HH27, HH29, HH30, HH31, HH32, HH33, HH34, HH35, HH36, HH37, HH38, HH39, HH40, HH41, HH42, HH43, HH44, HH45, HH46, HH47, HH48, HH49, HH50, HH51, HH52, HH53, HH54, HH55), 1 public building (PC01), 1 well water (WH03)
ND	ND	HH28, RC05, RH01, RS03, WC01, WC02, WC03, WC04, WH01, WH02, WH04, WH05

*First letter of isolate name indicates building type/location: H, hospital; R, residence; W, school using well water; P, large public building. Second letter indicates sample collection location; H, hot water tap; C, cold water tap; D, water heater drain valve; S, shower. Numerals 1–10 indicate clinical strains. ND, not determined because of lack of *L. pneumophila*–specific alleles or insufficient genome coverage; ST, sequence type. †New sequence types from this study submitted to European Working Group for Legionella Infections database (http://www.ewgli.org).

*L. pneumophila* isolates from clinical and water samples belonged to several STs (Table 2). Of serogroup 1 isolates, all belonged to STs 1, 44, 159, 192, 211, 213, or 222 or to a previously uncharacterized ST that we submitted to the European Working Group for *Legionella* Infections database (http://www.ewgli.org) and that has now been designated as ST2513. Serogroup 6 isolates all belonged to a previously uncharacterized ST that has now been designated as ST2518. Most hospital isolates belonged to ST2518, and isolates originating from residential tap water belonged primarily to ST192. Only ST1 was represented by both clinical and water isolates, specifically, 3 clinical isolates, 3 isolates from hospital tap water, and 1 isolate from residential tap water.

When classified according to SNP similarity, isolates formed distinct clades that were generally consistent with the ST classification (Figure). The ST1 clade varied by 2–1,062 SNPs, and isolates varied from the reference Paris strain by 371–505 SNPs. In particular, clinical isolate C3 shared the highest degree of similarity with Flint tap water isolates (38–46 SNPs). Some degree of variation is expected to be associated with variability in sequencing accuracy because the 3 clinical isolates that were sequenced in duplicate on multiple MiSeq lanes differed from their replicate by 0–10 SNPs. Several other distinct clades emerged in which water isolates were grouped primarily by building type. A large clade of ST2518 isolates included most samples from the hospital, 1 sample from well water, and 1 sample from a large public building. Another clade contained only isolates originating from Flint residence water samples belonging to ST192. The SNP results were confirmed by phylogenetic analysis and average nucleotide identity comparison (Appendix 1 Figures 1–3; Appendix 2).

**Figure:**
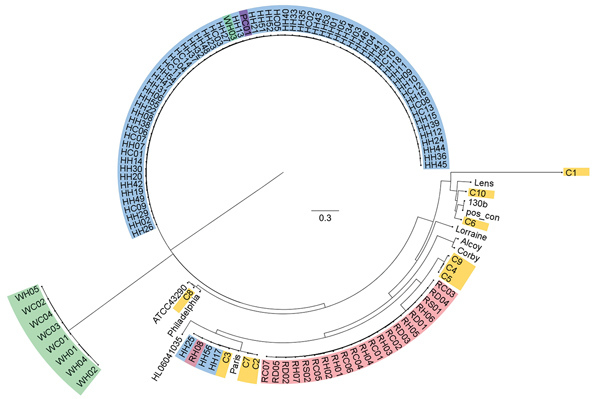
Single-nucleotide polymorphism (SNP) analysis of isolates from study of *Legionella pneumophila* in tap water, Flint, Michigan, USA. Analysis was conducted in kSNP3.0 (https://sourceforge.net/projects/ksnp/) and visualized by using FigTree 14.3 (https://github.com/rambaut/figtree/releases/tag/v1.4.3). Isolate sources: yellow, clinical samples; blue, hospital water; red, residence water; purple, public building water; green, buildings supplied by well water. With the exception of buildings supplied by well water, all buildings were serviced by Flint municipal water. Reference strains are detailed in Appendix 1 Table 2. Scale bar indicates nucleotide substitutions per site.

The STs of 8 isolates derived from well water could not be determined because *L. pneumophila*–specific alleles were absent, suggesting that the isolates were mistakenly phenotypically characterized as *L. pneumophila* on the basis of colony morphology. Average nucleotide identity values comparing these isolates with the positive control *L. pneumophila* strain (130b) were 62.645%–62.969%, whereas average nucleotide identity values of a single species are generally >95% (*21*). These 8 isolates seem to be most closely related to *L. taurinensis*, *L. rubrilucens*, or *L. erythra*, because the 16S rRNA genes extracted from these genomes shared >99% nt similarity to all 3 species.

## Discussion

When considered per capita, the Legionnaires’ outbreaks in Genesee County are among the largest in US history. However, to our knowledge, few clinical sputum isolates were collected or preserved from these outbreaks; for most cases, only urine-antigen testing was conducted. A common problem in the United States is reliance on urine-antigen testing and lack of collection of clinical *Legionella* isolates; these practices unfortunately limit the ability to track sources of infection, learn from past outbreaks, and prevent future outbreaks (*22*,*23*). Among the clinical sputum isolates that were sent to Genesee County laboratories during the outbreaks, none were from patients residing in homes serviced by Flint water (S. Lyon-Callo, MDHHS, pers. comm., 2018 Apr 5); thus, direct examination of potential residential exposure is not possible from this study. Given that 68% of patients’ residences were confirmed to not have been serviced by Flint water (*3*), the potential exists that a portion of the remaining 32% had some residential exposure in Flint.

Another challenge of tracking sources of Legionnaires’ disease is limited availability of water isolates. Given that the outbreaks were not publicly announced until 3 months after the conclusion of the second outbreak (January 2016), few environmental specimens were collected or preserved when the outbreaks were occurring. Analysis of any other water isolates that might exist from the time of the crisis would be valuable for learning more about this outbreak. MDHHS reported that 106 environmental *Legionella* specimens were retained at a Flint hospital but were not submitted to the State Health Department as had been requested (Sarah Lyon-Callo, MDHHS, pers. comm., 2019 Apr 30). Thus, a more definitive study of environmental sources of the outbreaks is not possible without a wider collection of clinical and environmental isolates.

Our study provides a survey of the landscape of genetic diversity among *Legionella* isolates collected from tap water from a range of building types served by the Flint drinking water distribution system over the 1-year period after the switch back to DWSD water. We compared these isolates with clinical isolates and with isolates from tap water of neighboring water systems never served by the Flint River or DWSD. Although it was not possible to collect water isolates during the actual outbreaks, previous studies have demonstrated that a single strain of *L. pneumophila* can colonize buildings and persist over multiple years (*24*–*26*). Thus, it is reasonable to assume that water isolates collected in 2016 were probably representative of strains colonizing building water systems over the previous months or even years.

Our study provides reasonable evidence that plumbing served by the Flint drinking water system was colonized by strains of *L. pneumophila* capable of causing Legionnaires’ disease, particularly serogroup 1 and ST1. Although no epidemiologic links have been made between clinical cases and cooling tower exposures in these outbreaks, direct or indirect use of tap water (e.g., via feed to cooling towers) is possible. High degrees of similarity (2–1,062 SNPs) were noted between the ST1 isolates of clinical and water origin, a finding that was consistent with phylogenetic and average nucleotide identity analysis (Appendix 1 Figures 1–3; Appendix 2). The highest degree of similarity between clinical and water isolates was between C3 and RH08 (38 SNPs), HH25 (40 SNPs), HH17 (45 SNPs), and HH56 (46 SNPs). C2 differed from water isolates by 1,053–1,062 SNPs, and C7 differed from water isolates by 1,041–1,049 SNPs. With the exception of 1 isolate (WH03), isolates obtained from tap water from buildings never served by the Flint River were markedly distinct from those originating from residences or hospitals in Flint as well as the clinical isolates. The low number of SNPs between replicate genomes sequenced in this study (0–10) suggests that the extent to which technical variation in whole-genome sequencing contributed to observed sequence variation is low. Previous studies have documented that although some Legionnaires’ disease outbreaks are characterized by *L. pneumophila* clinical strains that differ by as few as <5 SNPs, other outbreaks may differ by as many as 418 core SNPs (*27*). Thus, the SNP variability between water and clinical strains of ST1 in this study, particularly C3, is comparable to the documented range of variation in other outbreaks. In contrast, clinical strain C2 varied from the Paris reference strain by only 505, C3 by 371, and C7 by 491 SNPs. Therefore, ascertaining what level of SNP divergence between strains is demonstrative of a common source or virulent strain is challenging. Given the well-established pathogenicity of the Paris strain, the results are also suggestive of genomic similarity among virulent strains of *Legionella*. Regardless, the similarity between C3 and strains isolated from Flint tap water samples (38–46 SNPs) is notable.

ST1 water isolates were collected from taps of a hospital and a residence, indicating that this ST seems to have been somewhat widespread in the water distribution system, spanning multiple Flint buildings. However, the presence of several distinct phylogenetic clades of *L. pneumophila* isolated from Flint water systems further demonstrates that a single strain of *L. pneumophila* did not dominate the system citywide. We hypothesize that this finding is likely the result of conditions favorable to *Legionella* growth, which we previously documented in the Flint system (*7*), facilitating the proliferation of multiple strains of *L. pneumophila* in different buildings and parts of the system. Similarly, the broad distribution of clinical isolates across 7 STs supports the hypothesis that any waterborne exposures that resulted in Legionnaires’ disease could hypothetically have originated from a diverse array of *L. pneumophila* strains and exposure sources.

All clinical isolates characterized in this study belonged to *L. pneumophila* serogroup 1, which is identified as the cause of >57% of reported Legionnaires’ cases in the United States (*6*). ST1 (belonging to serogroup 1) has been widely implicated in Legionnaires’ outbreaks worldwide, including outbreaks in France (*28*), China (*29*), Germany (*30*), Canada (*31*), and the United States (*32*). In the United States, ST1 is thought to be both the most common cause of sporadic Legionnaires’ disease cases and the most common waterborne ST found in potable and nonpotable water (*32*). ST1 isolates are highly conserved at the nucleotide level (*33*), making it challenging to link clinical cases with environmental sources because of the prevalence of ST1 and lack of genetic variability.

Water isolates belonging to serogroup 6, all classified as ST2518, were widespread in samples collected from a Flint hospital in March 2016. A study of *L. pneumophila* isolates collected from Flint tap water in September and October 2016 also found that serogroup 6 isolates were widespread in residential premise plumbing water samples, although these isolates all belonged to STs 367 and 461 (*34*). Byrne and colleagues found that serogroup 6 strains were at least as infectious for macrophages as a known virulent laboratory strain, emphasizing the potential for Legionnaires’ disease to be caused by strains other than serogroup 1 (*34*), although more research is needed to confirm the relevance of serogroup 6 strains for human infectivity. In our study, none of the clinical strains available for analysis were serogroup 6.

It is noteworthy that 19% of hot water and 12% of cold water taps were positive for culturable *L. pneumophila*. Although *L. pneumophila* typically multiplies at 25°C–37°C (*35*) and prospers in hot water plumbing systems (*36*), it has also been widely documented in cold water taps; one molecular analysis–based study found that as many as 47% of surveyed cold water taps were positive for genes specific to *L. pneumophila* serogroup 1 (*37*).

When MDHHS recently conducted an epidemiologic characterization of the Genesee County Legionnaires’ disease cases recorded in 2014 and 2015, although a lack of clinical isolates hampered a comprehensive investigation, they found that exposures that occurred at 1 Flint hospital potentially explained most cases (*1*–*3*). Our study provides complementary whole-genome sequencing–based characterization of clinical isolates and tap water *L. pneumophila* isolates collected after the Flint outbreaks. Notably, we found a high degree of similarity between 4 water isolates originating from Flint tap water and 3 regional clinical strains known to cause Legionnaires’ disease. Our study also established that a variety of *L. pneumophila* strains were culturable from Flint tap water and that they tended to cluster genetically by residence versus hospital origin. Likewise, we found notable diversity among clinical strains, spanning 7 STs. Thus, multiple *L. pneumophila* strains were associated with the Flint 2014–2015 Legionnaires’ outbreaks, potentially resulting from multiple sources of exposure, although further epidemiologic investigation is needed to identify whether multiple sources were involved and whether there were any common sources of exposure. Although we did not intend for this study to provide an epidemiologic analysis of precise sources of *Legionella* exposure for Legionnaires’ patients, our publicly available data could support such studies in the future.

Appendix 1 Detailed description of the materials and methods used in whole-genome analysis of *Legionella pneumophila* in tap water, Flint, Michigan, USA.

Appendix 2 Pairwise average nucleotide identity values among *Legionella pneumophila* isolates. Values were calculated as previously described by Goris et al. 2007.
