# Supplementary material for: Comparison of Whole-Genome Sequences of Legionella pneumophila in Tap Water and in Clinical Strains, Flint, Michigan, USA, 2016
Source: Emerg Infect Dis. 2019 Nov;25(11):2013–20. doi: 10.3201/eid2511.181032 (PMC6810188; doi:10.3201/eid2511.181032)
Supplement: Appendix 1 — Detailed description of the materials and methods used in whole-genome analysis of Legionella pneumophila in tap water, Flint, Michigan, USA. [file 18-1032-Techapp-s1.pdf]

Article DOI: https://doi.org/10.3201/eid2511.181032

Comparison of Whole-Genome Sequences of *Legionella pneumophila* in Tap Water and in Clinical Strains, Flint, Michigan, USA, 2016

Appendix 1

Materials and Methods

Sample Collection and Preservation

Samples were collected from all taps into sterile polypropylene bottles (Nalgene, Rochester, NY) with 24 mg of sodium thiosulfate per liter added as a chlorine quenching agent. All samples were transported to the lab at room temperature and processed within ≈30 hours of sampling.

Aliquots (250–500 mL) were filter-concentrated onto a sterile 0.22 µm pore size mixed-cellulose ester membrane (Millipore, Billerica, MA) and resuspended in 5 mL sterile tap water before culturing *Legionella* according to standard methods (*1*). *L. pneumophila* colonies were streaked to isolation to obtain pure cultures. DNA was extracted from *Legionella* cultures by resuspending colonies in 50 µl of molecular grade water, freezing at −20°C, and rapidly thawing at 90°C for 10 minutes. Extracts were centrifuged at 10,000×g for 5 minutes to remove cell debris. Quantities of *Legionella* spp. and *L. pneumophila* gene markers from these samples have been published previously (*2*,*3*).

Whole Genome Sequence Analysis

16S rRNA gene sequences were extracted from sequence data using the Rapid Annotations Using Subsystem Technology server (*4*) and *Legionella* species assignments were determined via BLASTn of the sequence against the NCBI nucleotide database via the web server. Phylogenetic trees were generated using FastTree (*5*) based on extracted 16S rRNA gene sequences and 37 single-copy housekeeping genes in nucleotide space and amino acid space

using PhyloSift (*6*). ANI was calculated as previously described (*7*) and SNPs were identified using kSNP3.0 (*8*) with maximum likelihood estimation. Nine known *L. pneumophila* genomes associated with previous LD outbreaks were included in the analysis as reference strains for comparison (Table S2).

Sequence-based typing was performed targeting the *flaA*, *pilE, asd, mip, mompS, proA*, and *neuA* alleles (*9*) using the mompS tool (*10*). This bioinformatics tool facilitates backward compatibility of whole genome sequence typing by ensuring that the *momp*S allele, of which *L. pneumophila* can carry multiple non-identical copies, is typed based on the copy of the gene that would be identified by the traditional PCR-based typing scheme. STs were identified from allele profiles using the European Working Group for *Legionella* Infections database for *L. pneumophila*.

Serogroup Analysis

L. pneumophila isolate genomes belonging to serogroup 1 were identified via detection of the *wzm* gene (*11*) in whole genomes using BLAST with a minimum nucleotide identity of 98% and e-value of 1e-5. DNA sequence-based classifications were verified and unknown serogroups were determined using direct fluorescent antibody staining with FITC-conjugated antibodies (m-TECH, Milton, GA). To address problems with non-specific binding when stained cells were prepared according to manufacturer instructions, the protocol was modified as follows: isolates grown in buffered yeast extract broth (per liter: 10 g yeast extract, 1 g α ketoglutaric acid, 10 g 2-(carbamoylmethylamino)ethanesulfonic acid, 0.4 g L-cystine monohydrochloride, 0.25 g ferric pyrophosphate) were centrifuged at 5,000xg and resuspended in 1X phosphate buffered saline (PBS). To separate 25 µl aliquots of cells suspended in PBS, 5 µl of each FITC-conjugated antibodies were added and the suspension was incubated at 20°C for 30 minutes. Cells were washed with 1X PBS three times, then viewed with an AxioSkop2 plus fluorescence microscope (Carl Zeiss Microscopy, Oberkocken, Germany).

References

1. United States Centers for Disease Control and Prevention. Procedures for the Recovery of *Legionella* from the Environment. 2005.

2. Rhoads WJ, Garner E, Ji P, Zhu N, Parks J, Schwake DO, et al. Distribution System Operational Deficiencies Coincide with Reported Legionnaires' Disease Clusters in Flint, Michigan. Environ Sci Technol. 2017;51:11986–95. PubMed https://doi.org/10.1021/acs.est.7b01589

3. Schwake DO, Garner E, Strom OR, Pruden A, Edwards MA. *Legionella* DNA Markers in Tap Water Coincident with a Spike in Legionnaires' Disease in Flint, MI. Environ Sci Technol Lett. 2016;3:311–5. https://doi.org/10.1021/acs.estlett.6b00192

4. Aziz RK, Bartels D, Best AA, DeJongh M, Disz T, Edwards RA, et al. The RAST Server: rapid annotations using subsystems technology. BMC Genomics. 2008;9:75. PubMed https://doi.org/10.1186/1471-2164-9-75

5. Price MN, Dehal PS, Arkin AP. FastTree 2—approximately maximum-likelihood trees for large alignments. PLoS One. 2010;5:e9490. PubMed https://doi.org/10.1371/journal.pone.0009490

6. Darling AE, Jospin G, Lowe E, Matsen FA IV, Bik HM, Eisen JA. PhyloSift: phylogenetic analysis of genomes and metagenomes. PeerJ. 2014;2:e243. PubMed https://doi.org/10.7717/peerj.243

7. Goris J, Konstantinidis KT, Klappenbach JA, Coenye T, Vandamme P, Tiedje JM. DNA – DNA hybridization values and their relationship to whole-genome sequence similarities. 2017;57:81–91.

8. Gardner SN, Slezak T, Hall BG. kSNP3.0: SNP detection and phylogenetic analysis of genomes without genome alignment or reference genome. Bioinformatics. 2015;31:2877–8. PubMed https://doi.org/10.1093/bioinformatics/btv271

9. Gaia V, Fry NK, Afshar B, Lu PC, Etienne J, Peduzzi R, et al. Consensus Sequence-Based Scheme for Epidemiological Typing of Clinical and Environmental Isolates of *Legionella pneumophila*. 2005;43(5):2047–52.

10. Gordon M, Yakunin E, Valinsky L, Chalifa-Caspi V, Moran-Gilad J; ESCMID Study Group for Legionella Infections. A bioinformatics tool for ensuring the backwards compatibility of *Legionella pneumophila* typing in the genomic era. Clin Microbiol Infect. 2017;23:306–10. PubMed https://doi.org/10.1016/j.cmi.2017.01.002

11. Mérault N, Rusniok C, Jarraud S, Gomez-Valero L, Cazalet C, Marin M, et al.; DELPH-I Study Group. Specific real-time PCR for simultaneous detection and identification of *Legionella pneumophila* serogroup 1 in water and clinical samples. Appl Environ Microbiol. 2011;77:1708–17. PubMed https://doi.org/10.1128/AEM.02261-10

Appendix Table 1. Summary of environmental and clinical *L. pneumophila* isolates subject to whole genome sequencing. All clinical isolates were provided by the Michigan Department of Health and Human Services. All clinical isolates are from patients who resided outside of Flint.

SampleID[a]	Year Collected	Month Collected	Isolate Type	Building Type[b]	Sample Tap or Source	Flushed/ Stagnant	Water Source (April 2014-October 2015)	ST	SG
C1	2015		Clinical					159	1
C2*	2015		Clinical					1	1
C3*	2015		Clinical					1	1
C4	2015		Clinical					213	1
C5	2015		Clinical					213	1
C6	2015		Clinical					44	1
C7	2015		Clinical					1	1
C8	2015		Clinical					211	1
C9*	2015		Clinical					222	1
C10	2015		Clinical					2513	1
HC01	2016	March	Water	Hospital	Cold	Stagnant	Flint	2518	6[d]
HC02	2016	March	Water	Hospital	Cold	Stagnant	Flint	2518	6[d]
HC03	2016	March	Water	Hospital	Cold	Stagnant	Flint	2518	6[d]
HC04	2016	March	Water	Hospital	Cold	Stagnant	Flint	2518	6[d]
HC05	2016	March	Water	Hospital	Cold	Stagnant	Flint	2518	6[d]
HC06	2016	March	Water	Hospital	Cold	Stagnant	Flint	2518	6[d]
HC07	2016	March	Water	Hospital	Cold	Stagnant	Flint	2518	6[d]
HC08	2016	March	Water	Hospital	Cold	Stagnant	Flint	2518	6[d]
HC09	2016	March	Water	Hospital	Cold	Stagnant	Flint	2518	6[d]
HC10	2016	March	Water	Hospital	Cold	Stagnant	Flint	2518	6[d]
HC11	2016	March	Water	Hospital	Cold	Stagnant	Flint	2518	6[d]
HC12	2016	March	Water	Hospital	Cold	Stagnant	Flint	2518	6[d]
HC13	2016	March	Water	Hospital	Cold	Stagnant	Flint	2518	6[d]
HC14	2016	March	Water	Hospital	Cold	Stagnant	Flint	2518	6[d]
HH01	2016	March	Water	Hospital	Hot	Stagnant	Flint	2518	6[c]
HH02	2016	March	Water	Hospital	Hot	Stagnant	Flint	2518	6[d]
HH03	2016	March	Water	Hospital	Hot	Stagnant	Flint	2518	6[d]
HH04	2016	March	Water	Hospital	Hot	Stagnant	Flint	2518	6[c]
HH05	2016	March	Water	Hospital	Hot	Stagnant	Flint	2518	6[d]
HH06	2016	March	Water	Hospital	Hot	Stagnant	Flint	2518	6[d]
HH07	2016	March	Water	Hospital	Hot	Stagnant	Flint	2518	6[d]
HH08	2016	March	Water	Hospital	Hot	Stagnant	Flint	2518	6[d]
HH09	2016	March	Water	Hospital	Hot	Stagnant	Flint	2518	6[d]
HH10	2016	March	Water	Hospital	Hot	Stagnant	Flint	2518	6[d]
HH11	2016	March	Water	Hospital	Hot	Stagnant	Flint	2518	6[d]
HH12	2016	March	Water	Hospital	Hot	Stagnant	Flint	2518	6[d]
HH13	2016	March	Water	Hospital	Hot	Stagnant	Flint	2518	6[c]
HH14	2016	March	Water	Hospital	Hot	Stagnant	Flint	2518	6[d]
HH15	2016	March	Water	Hospital	Hot	Stagnant	Flint	2518	6[d]
HH16	2016	March	Water	Hospital	Hot	Stagnant	Flint	2518	6[d]
HH17	2016	March	Water	Hospital	Hot	Stagnant	Flint	1	1
HH18	2016	March	Water	Hospital	Hot	Stagnant	Flint	2518	6[d]
HH19	2016	March	Water	Hospital	Hot	Stagnant	Flint	2518	6[d]
HH20	2016	March	Water	Hospital	Hot	Stagnant	Flint	2518	6[d]
HH21	2016	March	Water	Hospital	Hot	Stagnant	Flint	2518	6[d]
HH22	2016	March	Water	Hospital	Hot	Stagnant	Flint	2518	6[d]
HH23	2016	March	Water	Hospital	Hot	Stagnant	Flint	2518	6[d]
HH24	2016	March	Water	Hospital	Hot	Stagnant	Flint	2518	6[d]
HH25	2016	March	Water	Hospital	Hot	Stagnant	Flint	1	1
HH26	2016	March	Water	Hospital	Hot	Stagnant	Flint	2518	6[d]
HH27	2016	March	Water	Hospital	Hot	Stagnant	Flint	2518	6[d]
HH28	2016	March	Water	Hospital	Hot	Stagnant	Flint	ND	ND
HH29	2016	March	Water	Hospital	Hot	Stagnant	Flint	2518	6[d]
HH30	2016	March	Water	Hospital	Hot	Stagnant	Flint	2518	6[d]
HH31	2016	March	Water	Hospital	Hot	Stagnant	Flint	2518	6[d]
HH32	2016	March	Water	Hospital	Hot	Stagnant	Flint	2518	6[d]
HH33	2016	March	Water	Hospital	Hot	Stagnant	Flint	2518	6[d]
HH34	2016	March	Water	Hospital	Hot	Stagnant	Flint	2518	6[d]
HH35	2016	March	Water	Hospital	Hot	Stagnant	Flint	2518	6[d]
HH36	2016	March	Water	Hospital	Hot	Stagnant	Flint	2518	6[d]
HH37	2016	March	Water	Hospital	Hot	Stagnant	Flint	2518	6[d]
HH38	2016	March	Water	Hospital	Hot	Stagnant	Flint	2518	6[d]
HH39	2016	March	Water	Hospital	Hot	Stagnant	Flint	2518	6[d]
HH40	2016	March	Water	Hospital	Hot	Stagnant	Flint	2518	6[d]

SampleID[a]	Year Collected	Month Collected	Isolate Type	Building Type[b]	Sample Tap or Source	Flushed/ Stagnant	Water Source (April 2014- October 2015)	ST	SG
HH41	2016	March	Water	Hospital	Hot	Stagnant	Flint	2518	6[d]
HH42	2016	March	Water	Hospital	Hot	Stagnant	Flint	2518	6[d]
HH43	2016	March	Water	Hospital	Hot	Stagnant	Flint	2518	6[d]
HH44	2016	March	Water	Hospital	Hot	Stagnant	Flint	2518	6[d]
HH45	2016	March	Water	Hospital	Hot	Stagnant	Flint	2518	6[d]
HH46	2016	March	Water	Hospital	Hot	Stagnant	Flint	2518	6[d]
HH47	2016	March	Water	Hospital	Hot	Stagnant	Flint	2518	6[d]
HH48	2016	March	Water	Hospital	Hot	Stagnant	Flint	2518	6[d]
HH49	2016	March	Water	Hospital	Hot	Stagnant	Flint	2518	6[d]
HH50	2016	March	Water	Hospital	Hot	Stagnant	Flint	2518	6[d]
HH51	2016	March	Water	Hospital	Hot	Stagnant	Flint	2518	6[c]
HH52	2016	March	Water	Hospital	Hot	Stagnant	Flint	2518	6[d]
HH53	2016	March	Water	Hospital	Hot	Stagnant	Flint	2518	6[d]
HH54	2016	March	Water	Hospital	Hot	Stagnant	Flint	2518	6[d]
HH55	2016	March	Water	Hospital	Hot	Stagnant	Flint	2518	6[d]
HH56	2016	March	Water	Hospital	Hot	Stagnant	Flint	1	1
PC01	2016	March	Water	Public building	Cold	Stagnant	Flint	2518	6[c]
RC01	2016	June	Water	Residence	Cold	Stagnant	Flint	192	1
RC02	2016	June	Water	Residence	Cold	Flushed	Flint	192	1
RC03	2016	August	Water	Residence	Cold	Stagnant	Flint	192	1
RC04	2016	June	Water	Residence	Cold	Stagnant	Flint	192	1
RC05	2016	June	Water	Residence	Cold	Stagnant	Flint	2514[c]	1
RC06	2016	June	Water	Residence	Cold	Stagnant	Flint	192	1
RC07	2016	June	Water	Residence	Cold	Flushed	Flint	192	1
RD01	2016	June	Water	Residence	HWHDV	NA	Flint	192	1
RD02	2016	June	Water	Residence	HWHDV	NA	Flint	192	1
RD03	2016	June	Water	Residence	HWHDV	NA	Flint	192	1
RD04	2016	June	Water	Residence	HWHDV	NA	Flint	192	1
RD05	2016	June	Water	Residence	HWHDV	NA	Flint	192	1
RH01	2016	June	Water	Residence	Hot	Flushed	Flint	ND	1
RH02	2016	June	Water	Residence	Hot	Stagnant	Flint	192	1
RH03	2016	June	Water	Residence	Hot	Flushed	Flint	192	1
RH04	2016	June	Water	Residence	Hot	Flushed	Flint	192	1
RH05	2016	June	Water	Residence	Hot	Stagnant	Flint	192	1
RH06	2016	June	Water	Residence	Hot	Stagnant	Flint	192	1
RH07	2016	June	Water	Residence	Hot	Stagnant	Flint	192	1
RH08	2016	August	Water	Residence	Hot	Stagnant	Flint	1	1
RS01	2016	June	Water	Residence	Shower	Stagnant	Flint	192	1
RS02	2016	August	Water	Residence	Shower	Stagnant	Flint	192	1
RS03	2016	June	Water	Residence	Shower	Stagnant	Flint	ND	ND
WC01	2016	March	Water	Well Water	Cold	Stagnant	well	NA	NA
WC02	2016	March	Water	Well Water	Cold	Stagnant	well	NA	NA
WC03	2016	March	Water	Well Water	Cold	Stagnant	well	NA	NA
WC04	2016	March	Water	Well Water	Cold	Stagnant	well	NA	NA
WH01	2016	March	Water	Well Water	Hot	Stagnant	well	NA	NA
WH02	2016	March	Water	Well Water	Hot	Stagnant	well	NA	NA
WH03	2016	March	Water	Well Water	Hot	Stagnant	well	2518	6[d]
WH04	2016	March	Water	Well Water	Hot	Stagnant	well	NA	NA
WH05	2016	March	Water	Well Water	Hot	Stagnant	well	NA	NA
pos_con			+ control					42	1
pos_con			+ control					42	1
neg_con			- control					NA	NA
neg_con			- control					NA	NA

[a]Isolates were named according to the following system: First letter indicates building type/location (H = hospital; R = residence; W = school using well water; p = large public building), second letter indicates sample collection location (hot water tap (H), cold water tap (C),water heater drain valve (D), shower (S)), followed by a unique numeric identifier. Clinical strains are denoted C1–10.
[b]Unless otherwise indicated, all buildings were serviced by Flint municipal water derived from the Flint River during the Flint Water Crisis
[c]Verified serogroup 6 using direct fluorescent antibody staining
[d]Presumed serogroup 6 based on direct fluorescent antibody staining of a phylogenetically diverse subset of isolates belonging to ST 2518
*indicates isolate was prepared and sequenced twice with consistent results as an additional control.
HWHDV = hot water heater drain valve; ST = sequence type; SG = serogroup
ND = could not be determined due to insufficient genome coverage; NA = not applicable

Appendix Table 2. Clinical reference strains selected for comparison to water isolates.

Sample ID	GenBank Accession Number	Origin	Serogroup	Sequence Type
LP Philadelphia	AE017354.1	USA	1	ST-136
LP ATCC 43290	CP003192.1	USA	12	ST-187
LP Alcoy	CP001828.1	Spain	1	ST-578
LP Corby	CP000675.2	UK	1	ST-51
LP Lens	CR628337.1	France	1	ST-15
LP 130b	FR687201.1	USA	1	ST-42
LP Paris	CR628336.1	France	1	ST-1
LP Lorraine	FQ958210.1	France	1	ST-47
LPHL06041035	FQ958211.1	France	1	ST-734

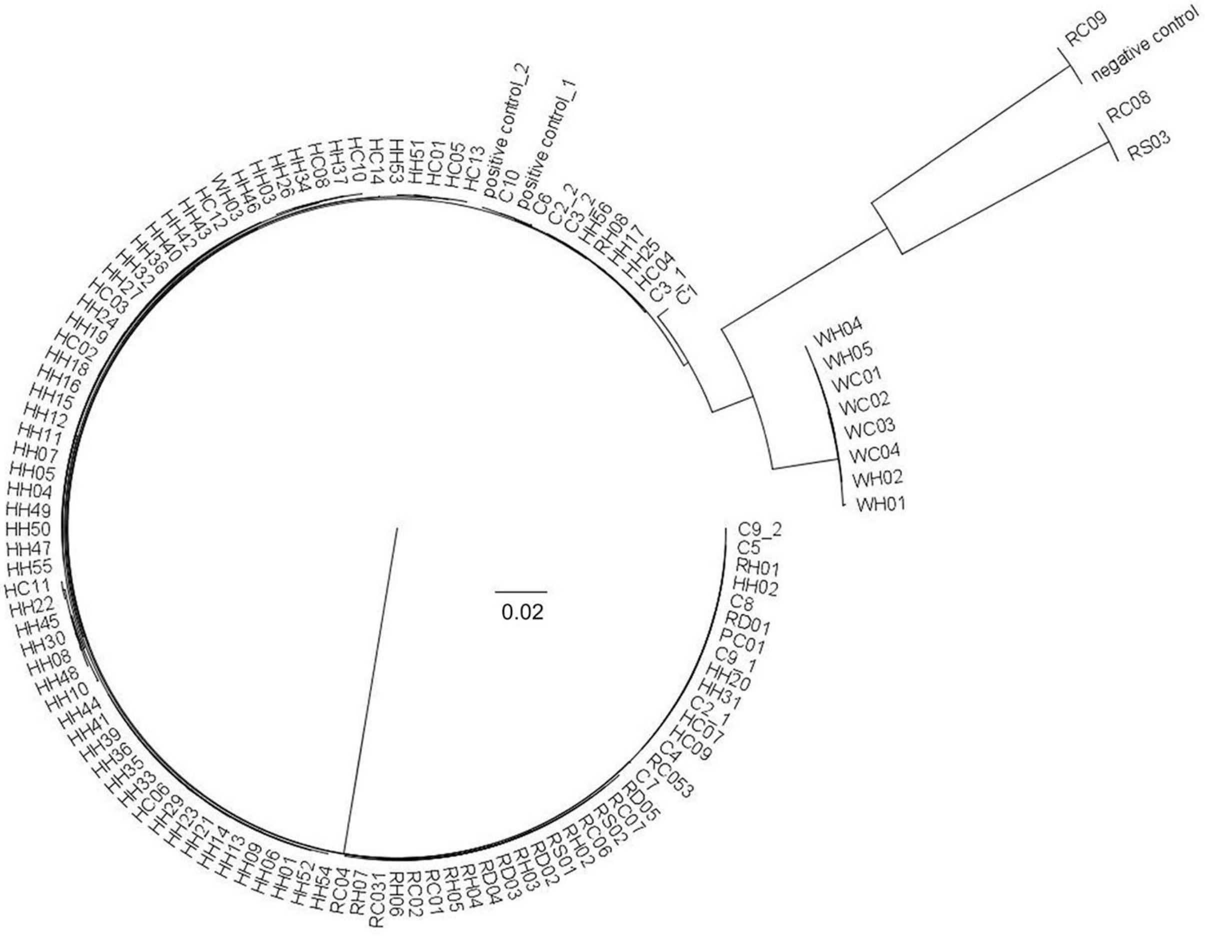

Appendix Figure 1. Phylogenetic tree generated using FastTree (Price et al. 2010) based on extracted 16S rRNA gene sequences using PhyloSift (Darling et al. 2014). Sample names appended with "_1" and "_2" represent isolates sequenced in duplicate on two different MiSeq runs.

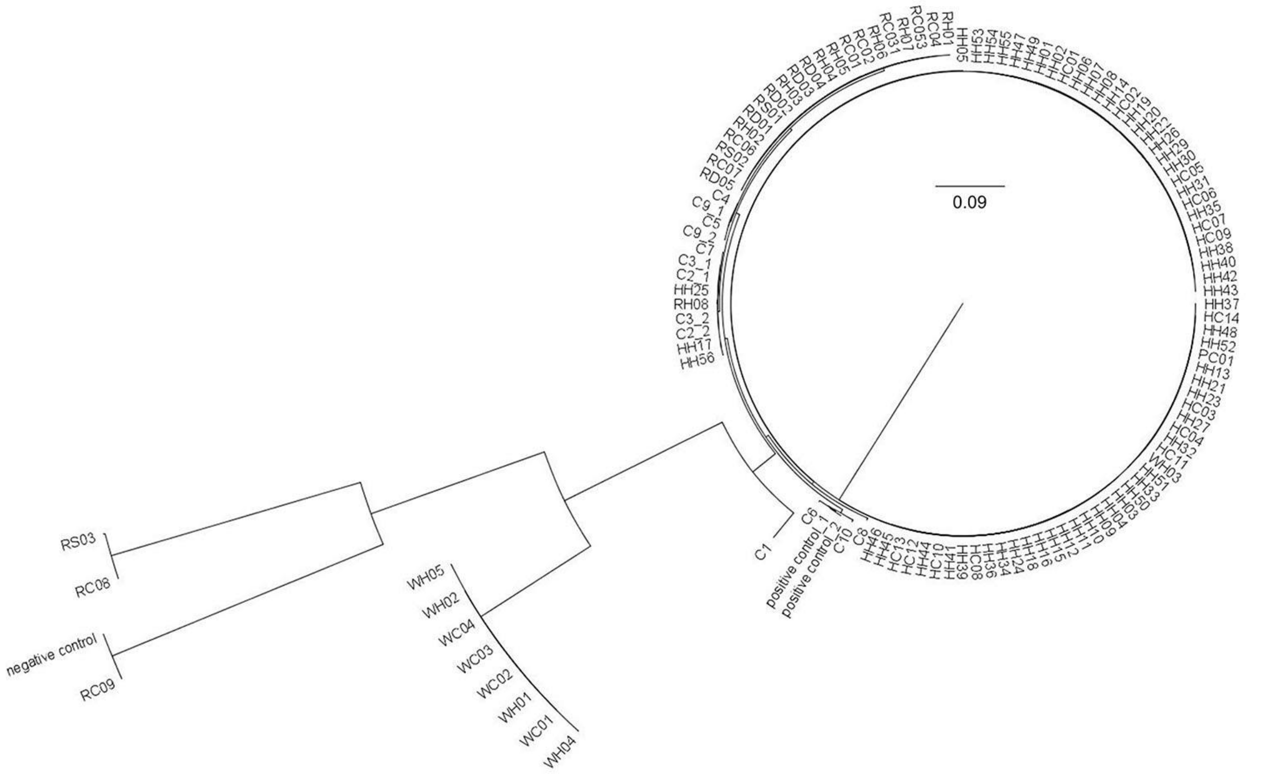

Appendix Figure 2. Phylogenetic tree generated using FastTree (Price et al. 2010) based on 37 single-copy housekeeping genes in amino acid space using PhyloSift (Darling et al. 2014). Sample names appended with "_1" and "_2" represent isolates sequenced in duplicate on two different MiSeq runs.

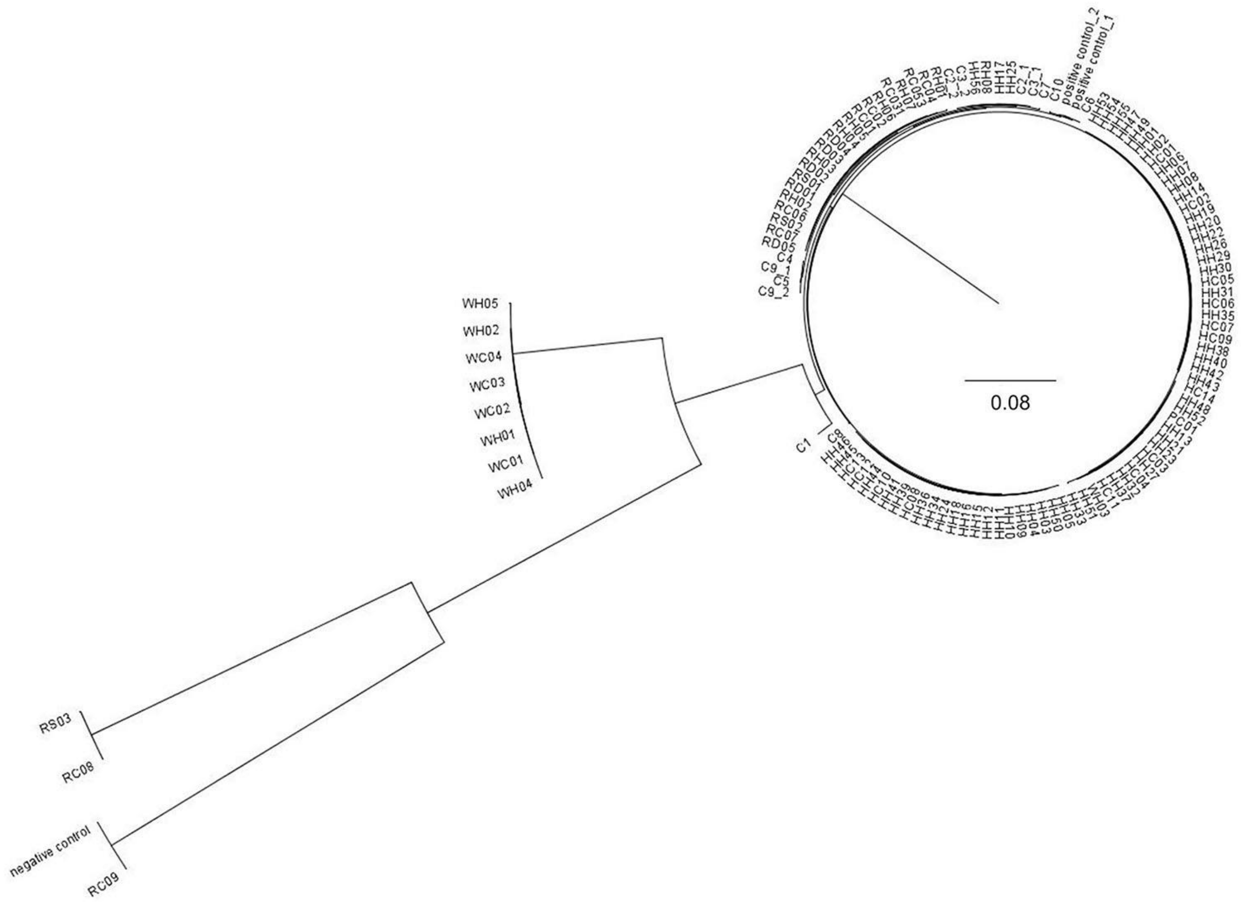

Appendix Figure 3. Phylogenetic tree generated using FastTree (Price et al. 2010) based on 37 single-copy housekeeping genes in nucleotide space using PhyloSift (Darling et al. 2014). Sample names appended with “_1” and “_2” represent isolates sequenced in duplicate on two different MiSeq runs.
